# Do alterations in pulmonary vascular tone result in changes in central blood volumes? An experimental study

**DOI:** 10.1186/s40635-021-00421-8

**Published:** 2021-12-17

**Authors:** Jaap Jan Vos, J. K. Götz Wietasch, Andreas Hoeft, Thomas W. L. Scheeren

**Affiliations:** 1 Department of Anesthesiology, University of Groningen, University Medical Center Groningen, Groningen, Netherlands; 2 Department of Anesthesiology, University of Bonn, Bonn, Germany

**Keywords:** Double-indicator transpulmonary thermo-dye dilution, Dogs, Pulmonary vascular tone, Blood volume, Hypoxia, Preload, Mean systemic filling pressure

## Abstract

**Background:**

The effects of selective pulmonary vascular tone alterations on cardiac preload have not been previously examined. Therefore, we evaluated whether changing pulmonary vascular tone either by hypoxia or the inhalation of aerosolized prostacyclin (PGI_2_) altered intrathoracic or pulmonary blood volume (ITBV, PBV, respectively), both as surrogate for left ventricular preload. Additionally, the mean systemic filling pressure analogue (Pmsa) and pressure for venous return (Pvr) were calculated as surrogate of right ventricular preload.

**Methods:**

In a randomized controlled animal study in 6 spontaneously breathing dogs, pulmonary vascular tone was increased by controlled moderate hypoxia (FiO_2_ about 0.10) and decreased by aerosolized PGI_2_. Also, inhalation of PGI_2_ was instituted to induce pulmonary vasodilation during normoxia and hypoxia. PBV, ITBV and circulating blood volume (Vd_circ_) were measured using transpulmonary thermo-dye dilution. Pmsa and Pvr were calculated post hoc. Either the Wilcoxon-signed rank test or Friedman ANOVA test was performed.

**Results:**

During hypoxia, mean pulmonary artery pressure (PAP) increased from median [IQR] 12 [8–15] to 19 [17–25] mmHg (*p* < 0.05). ITBV, PBV and their ratio with Vd_circ_ remained unaltered, which was also true for Pmsa, Pvr and cardiac output. PGI_2_ co-inhalation during hypoxia normalized mean PAP to 13 (12–16) mmHg (*p *< 0.05), but left cardiac preload surrogates unaltered. PGI_2_ inhalation during normoxia further decreased mean PAP to 10 (9–13) mmHg (*p* < 0.05) without changing any of the other investigated hemodynamic variables.

**Conclusions:**

In spontaneously breathing dogs, changes in pulmonary vascular tone altered PAP but had no effect on cardiac output, central blood volumes or their relation to circulating blood volume, nor on Pmsa and Pvr. These observations suggest that cardiac preload is preserved despite substantial alterations in right ventricular afterload.

## Background

**Fig. 1:**
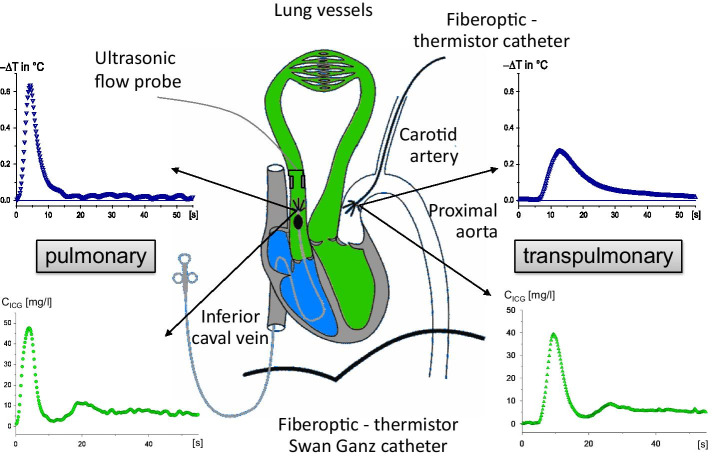
Schematic overview of the central blood volume compartment as determined by thermo-dye dilution. After injection of ice-cold indocyanine green in the right atrium, the dye dilution curves that are derived from the fiberoptic catheters placed in the pulmonary artery and ascending aorta, allow the calculation of PBV (green) and ITBV (blue and green combined. Modified from [5]

Pulmonary vascular tone (PVT) plays a central role in the regulation of cardiac preload and afterload, and may influence cardiac output (CO) by influencing venous return (VR) to the *left* ventricle (LV) and hereby determines LV preload. Simultaneously, PVT influences afterload of the *right* ventricle (RV). While VR was recognized as an important determinant of cardiac output (CO) decades ago by relating right atrial pressure (RAP) to LV output [1–4], it is important to consider that the RV receives blood from the *systemic* circulation, whereas the LV receives blood from the *pulmonary* circulation. The pulmonary blood volume compartment may therefore be regarded a surrogate measure of LV preload, which can be estimated reliably [5] by transpulmonary dilution as intrathoracic or pulmonary blood volume (ITBV and PBV, respectively, Fig. 1).The pulmonary circulation is a very compliant and low-pressure vascular bed [6, 7]. PVT can be altered artificially in both directions, e.g., by hypoxia or aerosolized prostacyclin (PGI_2_). Hypoxia increases PVT through hypoxic pulmonary vasoconstriction (HPV) [6], increasing pulmonary artery pressure (PAP). PGI_2_ administration instead, decreases PVT through relaxing of the vascular smooth muscle [8]. Interestingly though, the effects of selective alterations in PVT on the central blood volume compartment remains unknown to a large extent, as previous studies used isolated organ models only or solely focused on pressure-effects [9–12].

In addition, not only the pulmonary blood volume compartment itself, but also its distribution with the peripheral blood volume compartment might be influenced by alterations in PVT [13]. Therefore, the cumulative effect on the central blood compartment in terms of surrogate measures of (biventricular) cardiac preload, afterload and CO, remains elusive too.

Therefore, in this experimental study in spontaneously breathing dogs, we altered PVT in both directions by hypoxia and aerosolized prostacyclin, in order to gain more insight in the cumulative effects on ITBV and PBV as surrogate measures of LV preload. Since it is most likely that RV preload will be influenced as well, we calculated the pressure gradient between the right atrium and the more “upstream” side of the systemic circulation (mean systemic filling pressure (Pmsf) [2, 14–17], as a RV preload surrogate, in addition to mean PAP—resembling RV afterload.

Finally, to gain more insights in compensatory mechanisms in an intact circulation, the distribution between the PBV, ITBV and the (systemic) circulating blood compartment (Vd _circ_) has been studied as well.

## Methods

### Animals and instrumentation

Experiments were performed at the animal laboratory of the Department of Experimental Anesthesiology of the University of Düsseldorf, Germany. Reporting of the study was set up in accordance with ARRIVE 2.0 guidelines [18]. After approval from the local district governmental animal investigation committee (North-Rhine Westphalia in Dusseldorf, Germany; registered as Ref. 23.05-2303-84/96), six adult Foxhound dogs (3 males, 3 females, median age 38 months) were treated according to the principles of the National Institute of Health guidelines for animal care (NIH publication nr 86–23, revised 1985), the same dogs on which we previously reported [5].

The dogs were raised and housed in the Animal Research Laboratory of the Heinrich-Heine University (Düsseldorf, Germany). Relevant surgical procedures for preparation—performed several weeks before the experiments—have been described extensively before [5] and involved implanting an ultrasound transit-time flow probe (16–20 mm S-series with silicone shielded U-reflector, Transonic Systems, NY, USA) around the pulmonary artery for continuous recording pulmonary blood flow and cardiac output (CO_Transonic_). Two catheters were placed in the ascending aorta for blood sampling and arterial blood pressure measurement, and a fiberoptic thermistor probe (4F, Pulsiokath PV2024, Pulsion Medical Systems, Munich, Germany) was inserted to measure indocyanine green (ICG) plasma concentration and blood temperature. In addition, a 7F fiberoptic thermodilution catheter (Arrow International, Reading, MA) was introduced into a pulmonary artery via a dog’s hindlimb under fluoroscopy prior to each experiment. After completion of the experiments, the dogs were kept in the research facility until they died of natural causes.

### Measurements

Mean arterial blood pressure (MAP), mean PAP and central venous pressure (CVP) were measured at the level of the heart and simultaneously recorded on an eight-channel polygraph (model RS 3800, Gould, Cleveland, OH, USA) and a cassette data recorder (model XR-5000, TEAC®, Tokyo, Japan). Pulmonary capillary wedge pressure (PCWP) was determined intermittently. Partial arterial oxygen pressure (PaO_2_) and oxygen saturation (SaO_2_) were obtained by blood gas analysis (ABL3, Radiometer, Copenhagen, Denmark). CO_transonic_ was calibrated in vivo using the direct Fick principle using O_2_ uptake (Deltatrac II Metabolic Monitor), and the arterial to mixed venous oxygen content difference as measured by a galvanic cell (Lex-O2-CON-TL)—as described previously [19].

The determination of Vd _circ_, PBV and ITBV has been described in detail previously [5]. In short, blood volumes were determined using transpulmonary thermo-dye dilution by bolus injection of ice-cold ICG (< 5 °C, 0.2 mg kg^−1^). Here, ITBV represents the volume of blood between the injection site (right atrium) and recording site (ascending aorta), while PBV represents the volume of blood between the right atrium and aortic valve (Fig. 1). ITBV was calculated as: CO_transonic_ * mean transit time (mtt) of the ice-cold bolus between the respective injection and recording site. PBV was calculated similarly, yet the mtt was derived by deconvolution of the dye dilution curve that was based on a pulmonary transport function [20]. Vd_circ_ was calculated as: CO_transonic_ * mtt of the overall circulation in a 30 min time period, as fitted by an aortic dye dilution curve over a recirculation model. The ratios between central and systemic circulating blood volumes (ITBV/Vd _circ_ and PBV/Vd _circ_) were calculated afterwards.

Pmsf was estimated mathematically using a Pmsf analogue (Pmsa) [15, 21] offline using a validated algorithm [15] incorporating MAP, CO_Transonic_ and CPV. Pmsa was calculated using the formula:

${\text{Pmsa }} = \, a \, *{\text{ CVP }} + \, b \, *{\text{ MAP }} + \, c \, *{\text{ CO}}$. Here, *a* + *b* = 1 (*a* = 0.96 and *b* = 0.04, reflecting the contribution of venous and arterial compartments), *c* reflects an assessment of resistance and is determined on age, weight and height and was determined according to previous studies in dogs [22]. Pressure for venous return (Pvr) was calculated as: ${\text{Pvr }} = {\text{ Pmsa }}{-}{\text{ CVP}}$, heart efficiency (Eh) as: Eh = Pvr/Pmsa, and resistance for venous return (RVR) as: RVR = Pvr/CO.

### Experimental program

Anesthesia was performed using pentobarbital (20 mg kg^−1^ injection, followed by a continuous infusion of 4 mg kg^−1^ h^−1^). The dogs were breathing room air spontaneously.

**Fig. 2:**
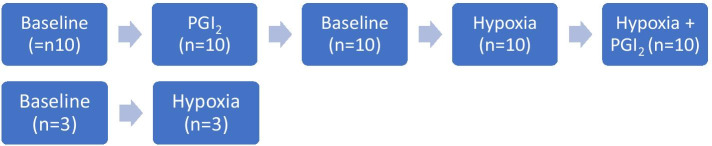
Schematic overview of the order of intervention and the number of interventions performed

We increased PVT by lowering inspiratory oxygen concentration by adding nitrogen (AGA Linde, Medical Gases) to a plastic hood fixed above the dog’s head and upper trunk. The flow rate was adjusted to reduce FiO_2_ to about 0.1. FiO_2_ was measured continuously via a gas probe fixed in the middle of the hood above the dog’s head (Capnomancy® Ultima SV, Datex-Engstrom, Helsinki, Finland). PVT was decreased by adding aerosolized prostacyclin (PGI_2_) to the inspiratory gases: 10 μg/ml crystalline PGI_2_ (Flolan®, GlaxoWellcome, Hamburg, Germany) solution was instilled into the chamber of an ultrasound nebulizer (Siemens Elema, Solna, Sweden) by an infusion pump (Perfusor, Braun, Melsungen, Germany), which was connected to the orifice of a tube, inserted into the trachea prior to this intervention to minimize absorption of PGI_2_ at the upper airways. Aerosolized PGI_2_ was inhaled during normoxia (FiO_2_ 0.21) or during hypoxia (FiO_2_ 0.1). The nebulizer gas flow (pressurized air and nitrogen) was adjusted (range 6–10 L min^−1^) to achieve and maintain a reduction in mean pulmonary arterial pressure of 10% below baseline. The mean dose of aerosolized PGI_2_-solution was approximately 7.5 µg kg^−1^ min^−1^. To assure hemodynamic stability, the dogs were observed for 30 min after introduction of the catheters. The intervention sequence started with PGI_2_ administration, followed by hypoxia and ended with PGI_2_ co-administration (*n* = 10). In three sequences, hypoxia was administered only, without PGI_2_ (co-) administration due to logistic reasons. Before each intervention, a separate baseline measurement was performed to serve as baseline for that particular intervention (Fig. 2).

Interventions lasted approximately 30 min each. Measurements were performed at the end of each intervention during steady state. There were no relevant data available to estimate effect sizes of interventions, so an a priori sample size calculation was not performed.

### Statistics

Statistical analysis was performed using SigmaPlot 13.0 (Systat Software, San Jose, USA). CO_Transonic_ values were normalized for body weight. Continuous data were assumed non-normally distributed. Analyses were performed pairwise: data from interventions were compared with preceding baseline measurements to account for within-subject variability. The Wilcoxon-signed rank test was used for pairwise comparisons. In case of three consecutive measurements, the Friedman ANOVA was used, and the Dunn’s test was applied for post hoc testing. Statistical significance was set at *P*-values < 0.05.

## Results

A total of 33 interventions were performed in 6 dogs on 13 different study days. PVT was increased by the administration of a hypoxic gas mixture, with FiO_2_ ranging from 0.08 to 0.12, which was temporarily combined with the inhalation of aerosolized PGI_2_ in ten of these interventions. In 10 interventions, PVT was solely reduced using inhaled aerosolized PGI_2_, without simultaneous administration of a hypoxic gas mixture. No adverse events occurred during any of the interventions.

**Fig. 3:**
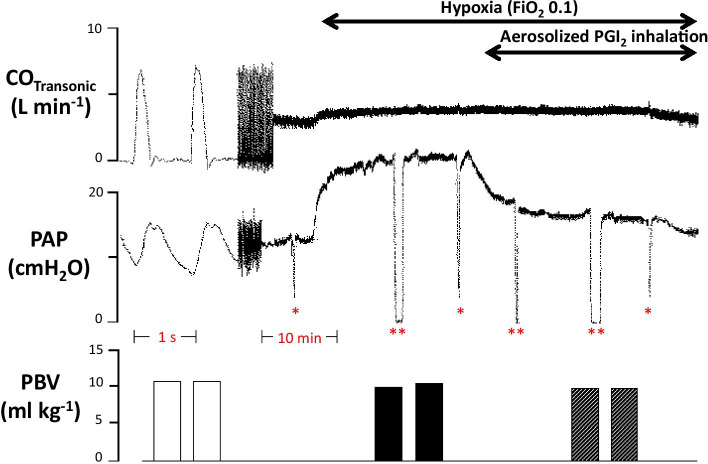
Original registration in an anesthetized spontaneously breathing dog. After 2 heart cycles, recording speed was reduced and tracings of CO_Transonic_ and pulmonary arterial pressure (PAP) were switched to mean values. At the end of each intervention, blood volumes were determined in duplicate. During hypoxia, PAP increased markedly, whereas pulmonary capillary wedge pressure (PCWP), and pulmonary blood volume (PBV) remained unchanged. *: PCWP was determined intermittently at three moments. **: a zero-adjustment was performed at three moments

A typical example is shown in Fig. 3.

### Hypoxia

During hypoxia mean PAP increased from a median (interquartile range) value of 12 (8–15) mmHg at normoxia to 19 (17–25) mmHg (*p* < 0.05; Table 1) during hypoxia. MAP increased from 85 (82–91) mmHg during normoxia, to 93 (90–97) mmHg during hypoxia (*p* < 0.05). SV decreased from 27 (25–33) mL during normoxia to 23 (19–27) mL during hypoxia (*p* < 0.05). There was no change in CO. heart rate (HR) increased from 90 (82–97) bpm during normoxia to 115 (102–130) bpm during hypoxia (*p* < 0.05). Neither CVP nor PCWP changed during hypoxia (Table 1).

**Table 1:**
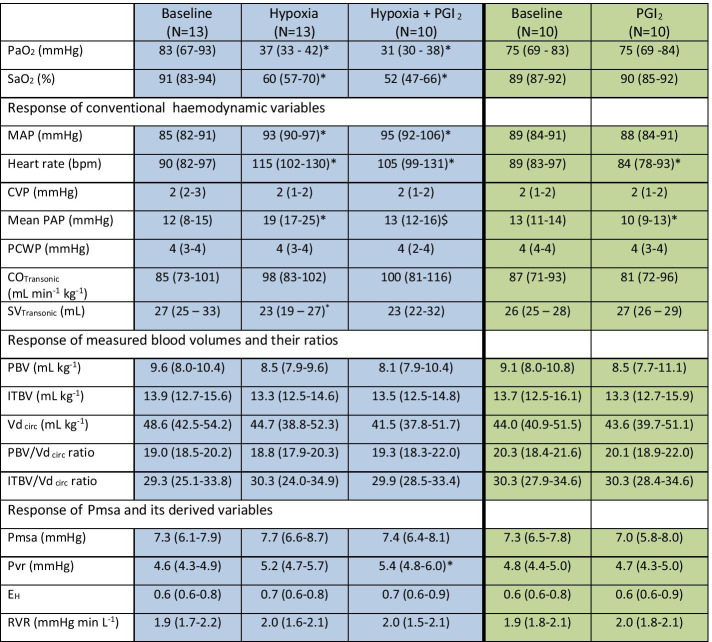
Hemodynamic changes in response to induced hypoxia and co-administration of inhaled PGI_2_, as well as the reaction to solitary administration of inhaled PGI_2_

Data are presented as median (interquartile range). PGI_2_, Aerosolized prostacyclin; PaO_2_, Arterial Partial Pressure of Oxygen; SaO_2_, Arterial Oxygen Saturation; Vd _circ_, Effective circulating blood volume; MAP, Mean Arterial Pressure; CVP, Central Venous Pressure; PAP, Pulmonary Artery Pressure; PCWP, Pulmonary Capillary Wedge Pressure (PCWP); CO_Transonic_, SV_Transonic_, Cardiac Output, Stroke Volume derived from Transonic Flow Probe; PBV, Pulmonary Blood Volume; ITBV, Intrathoracic Blood Volume; Pmsa, mean systolic pressure analogue; Pvr, driving pressure for venous return; E_H_, cardiac performance; RVR, resistance to venous return

^*^P < 0.05 vs baseline

^$^P < 0.05 Hypoxia vs hypoxia + PGI_2_

**Fig. 4:**
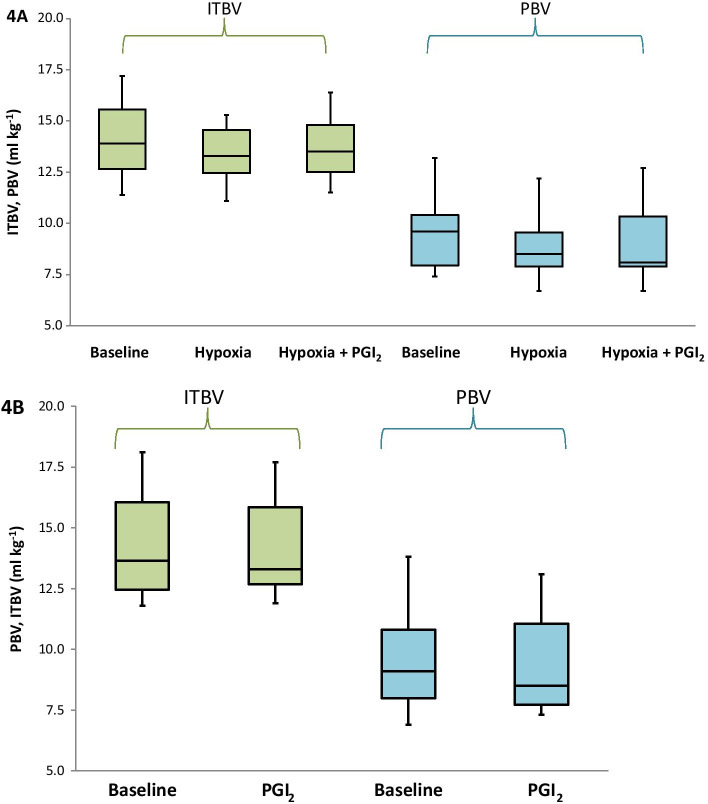
**A** Boxplot in which the absolute values of ITBV (green) and PBV (blue) are shown at baseline (*n* = 13), during hypoxia (*n* = 13), and during inhalation of aerosolized PGI_2_ (*n* = 10) while the dogs were still exposed to hypoxia. **B** Boxplot in which the absolute values of PBV (blue) and ITBV (green) are shown at baseline (*n* = 10) and during subsequent administration of PGI_2_ (*n* = 10)

There was no change in both PBV and ITBV, neither during normoxia, nor during hypoxia (Fig. 4 and Table 1). Moreover, both Vd_circ_, and the ratio between Vd_circ_ and PBV, and ITBV, respectively, remained unaltered as well as Pmsa and derived variables (Table 1).

### Inhaled aerosolized PGI_2_

Aerosolized PGI_2_ inhalation *under normoxia* decreased mean PAP from 13 (11–14) mmHg to 10 (9–13) mmHg, *p* < 0.05. HR decreased from 89 (83–97) bpm to 84 (78–93) bpm (*p* < 0.05). Other conventional hemodynamic variables remained unchanged after administering PGI_2_, as shown in Table 1.

There were no changes in PBV, ITBV, Vd_circ_ (Table 1, Fig. 4B), nor was there a change in V_D circ_ or its ratio with PBV or ITBV. This was also true for Pmsa and derived variables (Table 1).

Upon co-administration of aerosolized PGI_2_ *during hypoxia*, the increase in mean PAP was reversed (i.e., from a median value of 19 mmHg back to 13 mmHg, *p* < 0.05). MAP and HR increased to 95 (92–106) mmHg and 105 bpm, respectively, compared to baseline values (*p* < 0.05). Other conventional hemodynamic variables remained unaltered (Table 1).

There were again no changes in any of the measured blood volumes, neither did the ratio between either PBV or ITBV with Vd _circ_ change (Fig. 4A, Table 1). This was also true for the calculated values of Pmsa and its derived variables, except for a slight increase in Pvr.

## Discussion

In this experimental study in spontaneously breathing dogs, hypoxia substantially increased PVT. SV decreased, which was compensated by an increase in HR so that ultimately CO remained constant. Preload was maintained too, both for the LV (PBV, ITBV) and for the RV (Pvr) and the distribution of central and circulating blood volume remained unaltered. Therefore, ﻿it appears that an increase in PAP as a surrogate of RV afterload does not impair CO in healthy dogs, has no measurable effect on left and right cardiac preload, and does not alter the distribution of blood within the intrathoracic and systemic circulation. In addition, reducing PVT by endobronchial PGI_2_, neither changed CO, nor changed any of the surrogate measures of preload, and finally neither altered the distribution of blood between the central blood volume compartment and circulating blood volume compartment.

### Effects of hypoxia on the pulmonary circulation

HPV substantially increased mean PAP. HPV predominantly entails precapillary vascular smooth muscle contraction, and is a well-recognized phenomenon in mammals that helps to match regional perfusion and ventilation [7]. While being a protective native reflex, HPV may be particularly dangerous in subjects who cannot tolerate the associated (sudden) increase in PAP, e.g., those with reduced (right) ventricular function or an intracardiac shunt. E.g., pulmonary atelectasis with associated regional hypoxia—inducing HPV—may preserve systemic oxygenation at the cost of an increase in RV afterload [23].

We observed that the increase in mean PAP during hypoxia was associated with an increase in both HR and MAP. Likely, this observation can be attributed to an increased sympathetic tone [24]. The increase in HR allowed CO maintenance during hypoxia, despite a decrease in SV. It remains to be elucidated whether SV decreased secondary to the increase in afterload or hypoxia-induced myocardial ischemia (albeit no electrocardiographic signs of ischemia were noted). Most importantly, the increase in mean PAP following hypoxia was not accompanied by a change in PBV and ITBV, indicating that LV preload remained preserved—a subject that has not been investigated in an intact circulation before. Previous studies that were performed in isolated animal lung *preparations* were inconclusive as central blood volume was either reduced [11], preserved [12], or increased [25]—in response to HPV.

Secondarily, Pmsa and Pvr remained constant as well during hypoxia, suggesting that the increase in RV afterload did not affect RV preload. As Pmsa (resembling the net balance between systemic vascular tone and volume) remained constant, we conclude that hypoxia did not change systemic vascular tone and volume, and consequently did not change RV preload, as resembled by a constant Pvr. The unaltered heart efficiency (E_H_; Pvr divided by Pmsa) additionally shows that CO was maintained and could “overcome” VR in spite of the increase in RV afterload, with a maintained pressure gradient between the (right) heart and the returning venous blood. Hence, the resistance to venous return (RVR), in which CVP acts as an opposing pressure for generating CO, remained unaltered. In other words: despite the sudden hypoxia-induced increase in RV afterload, the heart was able to maintain RAP (or CVP) low, in order to preserve VR to the right heart.

It must be stressed that PBV and ITBV were considered as *surrogate* measures of cardiac preload and we did not record functional measures of LV preload, e.g., stroke volume variation. Still, we looked for *changes* in these blood volumes, and the net conclusion from the above-mentioned mechanisms predominantly support the hypothesis that RV and LV preload remained unaltered.

Finally, as we simultaneously investigated the influence of changes in PVT on Vd_circ_, we could demonstrate absence of impact on the distribution between PBV, ITBV and Vd_circ_. We speculate that increases in PVT can be “easily” countered by the high compliance of the pulmonary circulation.

### The effects of PGI_2_

PGI_2_ is a potent vasodilator that can be applied systemically (i.v.) and endobronchially (aerosolized PGI_2_) [26, 27]. After endobronchial administration, aerosolized PGI_2_ produces selective pulmonary vasodilation, i.e., PAP is selectively lowered without lowering systemic arterial pressure [28]. To the best of our knowledge, the effects of selective pulmonary vasodilation (e.g., by PGI_2_ administration) on blood volumes have not been studied before in an in vivo model with an intact circulation. In this study, we observed a minor effect of PGI_2_ inhalation on PAP and MAP without any effect on measures of central blood volume, Pmsa and Pvr. Interestingly however, PGI_2_ administration during hypoxia allowed mean PAP to return to pre-hypoxic values, suggesting that hypoxia “unmasks” the pulmonary vasodilatory effects of PGI_2_ and resolves HPV *despite* ongoing hypoxia: it may be argued that PGI_2_ has no further vasodilatory influence on PVT under normal conditions.

Additionally, we observed that the distribution between PBV, ITBV and Vd_circ_ were unaffected by *aerosolized* PGI_2_, which contrasts previous research where prostacyclines were administered *systemically* for malignant hypertension treatment in humans (*n* = 7) [28]. Although Pvr decreased subtly during PGI_2_ co-administration during hypoxia, which may suggest a very slight increase in RV preload, this observation is probably of negligible clinical significance. While SV was preserved, CO remained constant as well, although it may be speculated that if a larger sample size was obtained, the (trend-like) changes in CO following PGI_2_ inhalation may have turned statistically significant. Finally, it might be speculated that PGI_2_ inhalation directly causes a reduction in heart rate. To the best of our knowledge, there is no literature available to support this assumption.

## Study limitations

We have previously demonstrated that the applied instrumentation permits a reliable assessment of ITBV, PBV and Vd_circ_ [5], meaning that the applied technique can be regarded sufficiently sensitive to detect small changes in the measured blood volumes. Yet, it is important to stress that Vd_circ_ systematically underestimates total blood volume by about 40% [29] mainly due to incomplete mixing of the dye in more slowly perfused tissues. However, changes in Vd_circ_ can still be regarded reliable [5, 20]. Also, previous investigations either focused solely at pressure-effects, or were performed on perfused isolated organs [9, 10, 12] and did not allow assessing the effects of selective, solitary alterations in PVT on an intact circulation which we considered indispensable since it is the *systemic* circulation that determines VR. This implies that the complex interplay between cardiac preload and afterload can only be reliably assessed in an intact circulation.

In contrast, the use of a fixed FiO_2_ (about 0.10) and standardized PGI_2_ doses may be considered a limitation of the study, as the hemodynamic effects may be ‘dose’-dependent. It may be argued that during more severe hypoxia and/or higher dosages of PGI_2_, compensatory physiologic mechanisms might fail and would reveal a more pronounced hemodynamic perturbance, although the current observations do not demonstrate such a trend.

Since the spleen and hepatosplanchnic circulation function as a blood reservoir in dogs in case of sympathetic activation [30], blood volumes may be altered by hypoxia and aerosolized PGI_2_ inhalation. However, given that Vd_circ_ remained unaltered during the study, this effect—if there were any—is most likely negligible.

Our experiments took place while dogs were breathing spontaneously and observations can therefore not be extrapolated directly to mechanically ventilated subjects. Mechanical ventilation is associated with a shift of blood from the intra- to the extrathoracic compartment [31], which would intrinsically influence the results. To account for the effects of endotracheal intubation, a control group served for evaluation of time-related and spontaneous variations in pulmonary and systemic variables. In this control group, the dogs breathed air with and without an endotracheal tube, and no hemodynamic differences were observed (data not shown).

Finally, in the primary outcome variables of this study—i.e., the data on measured blood volumes—no statistically significant differences were found between the various interventions. It should be stressed that a relatively limited number of subjects was investigated, in which a limited number of interventions was performed. Even though the observed effect sizes between interventions appear to be very small, it may be true that a larger sample size would have resulted in (subtle) statistically significant differences between the various interventions.

## Conclusions

In this experimental study in spontaneously breathing dogs, hypoxia increased pulmonary vascular tone substantially. Cardiac output was maintained, owing to an increase in heart rate to compensate a decrease in stroke volume. Central blood volumes and their distribution with circulating blood volume, and the pressure for venous return remained unaltered.

PGI_2_ decreased pulmonary vascular tone substantially: CO, central blood volumes and their relation to circulating blood volume, as well as pressure for venous return remained unaltered.

These cumulative observations suggest that cardiac preload was preserved despite substantial alterations in pulmonary vascular tone, i.e., right ventricular afterload.

## Data Availability

The datasets used and/or analyzed during the current study are available from the corresponding author on reasonable request.

## Abbreviations

CO: Cardiac output
CVP: Central venous pressure
Eh: Heart efficiency
FiO_2_: Fraction of inspired oxygen (O_2_)
HPV: Hypoxic pulmonary vasoconstriction
ITBV: Intrathoracic blood volume
LV: Left ventricle
MAP: Mean arterial pressure
PaO_2_: Arterial partial oxygen pressure
PAP: Pulmonary artery pressure
PBV: Pulmonary blood volume
PCWP: Pulmonary capillary wedge pressure
PGI_2_: Aerosolized prostacyclin
Pmsa: Analogue of mean systemic filling pressure
Pmsf: Mean systemic filling pressure
Pvr: Pressure for venous return
PVT: Pulmonary vascular tone
RAP: Right atrial pressure
RV: Right ventricle
RVR: Resistance to venous return
SaO_2_: Arterial oxygen saturation
Vd_circ_: Circulating blood volume
VR: Venous return
